# Supplementary material for: Maternal Salivary miR-423-5p Is Linked to Neonatal Outcomes and Periodontal Status in Cardiovascular-High-Risk Pregnancies
Source: Int J Mol Sci. 2024 Aug 22;25(16):9087. doi: 10.3390/ijms25169087 (PMC11354562; doi:10.3390/ijms25169087)
Supplement: Supplementary file 1 [file ijms-25-09087-s001.zip › ijms-3124059-supplementary.pdf]

Supplementary

Figure S1 - EV-miRNA quality and integrity data

EV-miRNA quality and integrity data of ten representative samples (H: Healthy-H, Gingivitis-G and Periodontitis-P) assessed through *2100 Bioanalyzer RNA system* with the Pico Kit (Agilent Technologies, Santa Clara, CA, USA). Data are shown as electrophoresis run summary, electrophoresis graphs and RIN score.

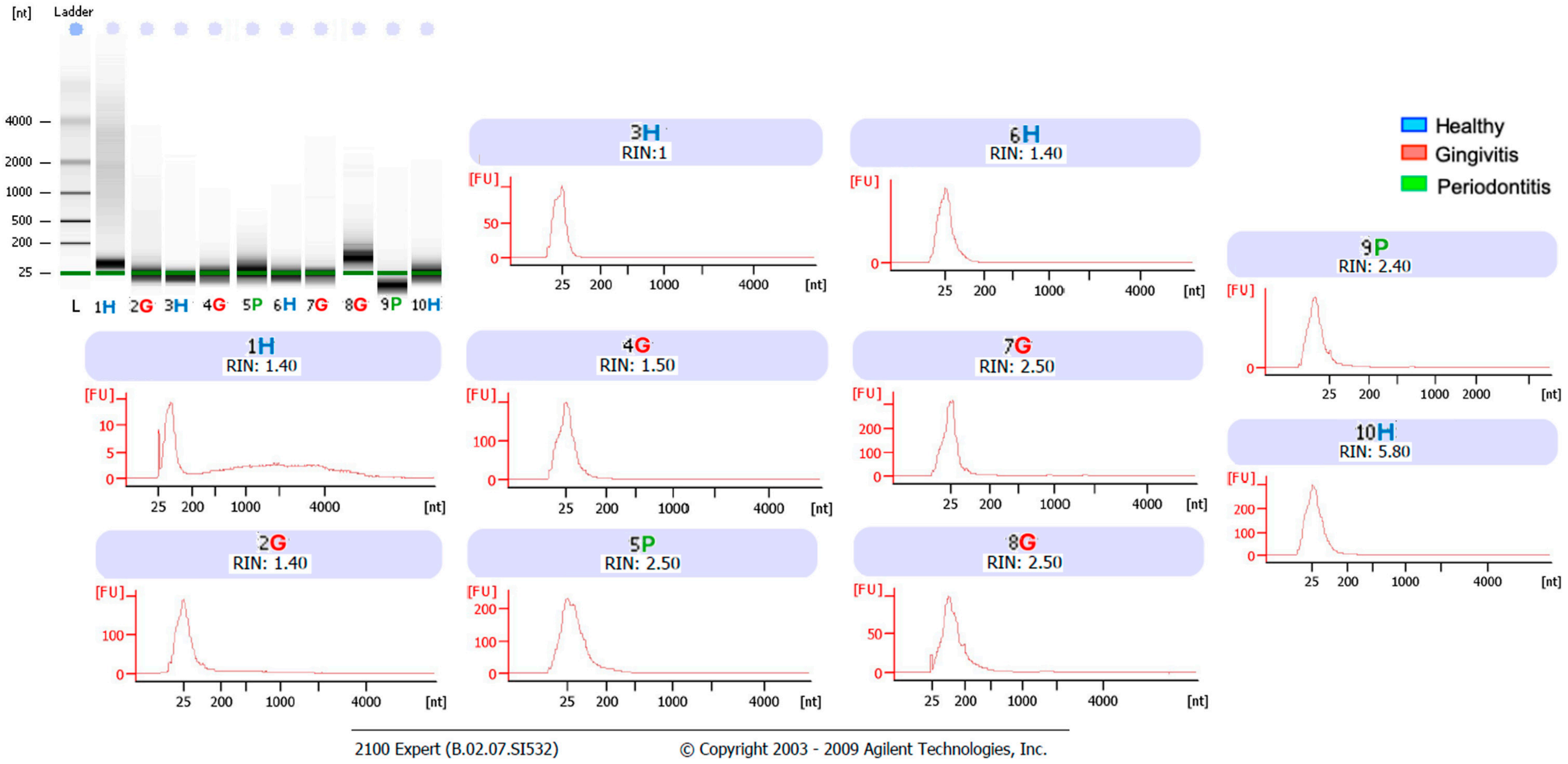
[nt]
Ladder
4000
2000
1000
500
200
25
L
1H
2G
3H
4G
5P
6H
7G
8G
9P
10H
Healthy
Gingivitis
Periodontitis
3H
RIN:1
6H
RIN: 1.40
9P
RIN: 2.40
1H
RIN: 1.40
4G
RIN: 1.50
7G
RIN: 2.50
10H
RIN: 5.80
2G
RIN: 1.40
5P
RIN: 2.50
8G
RIN: 2.50
[FU]
[nt]
2100 Expert (B.02.07.SI532)
© Copyright 2003 - 2009 Agilent Technologies, Inc.

Figure S2- miRNA cluster dendograms

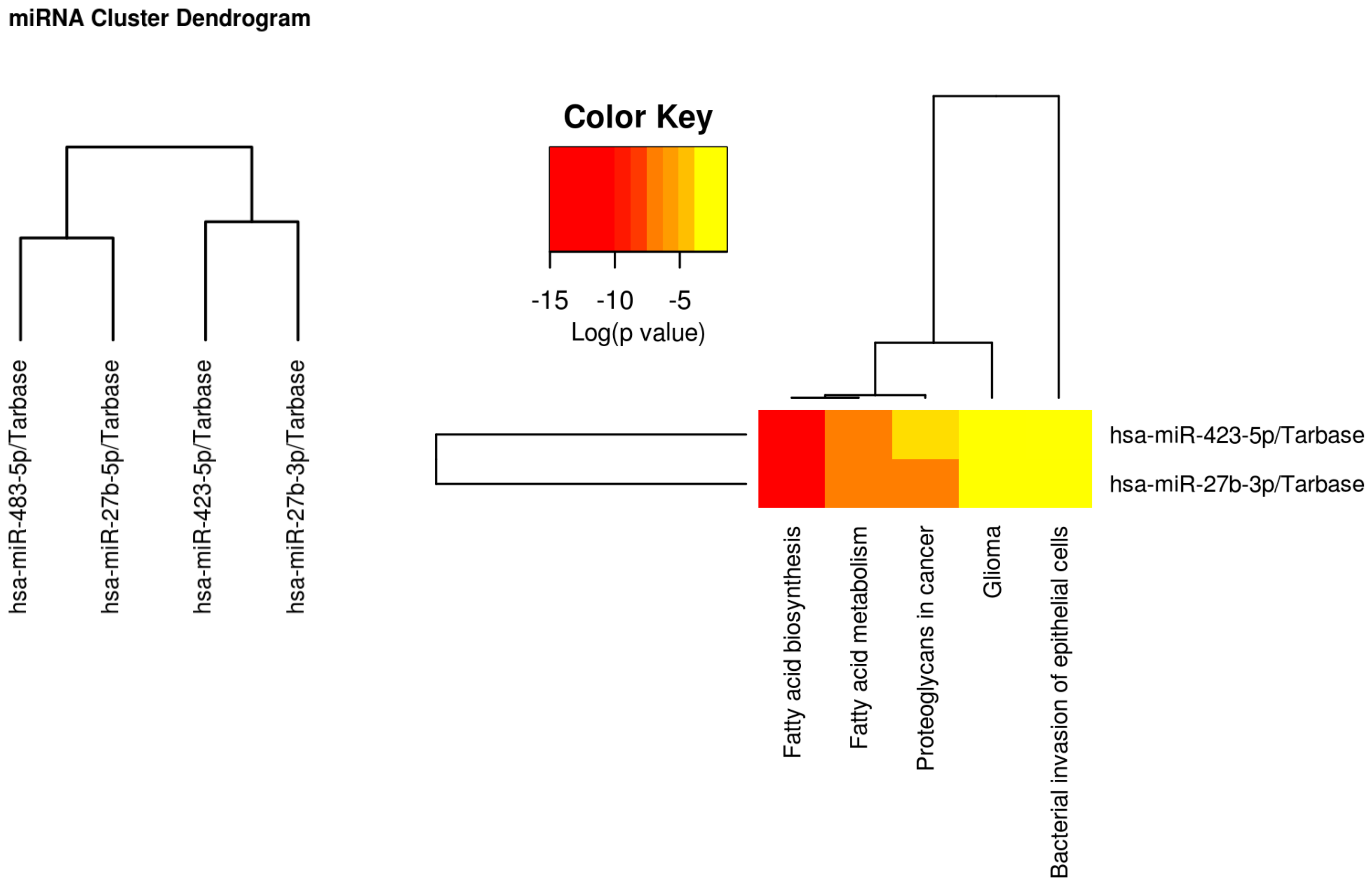

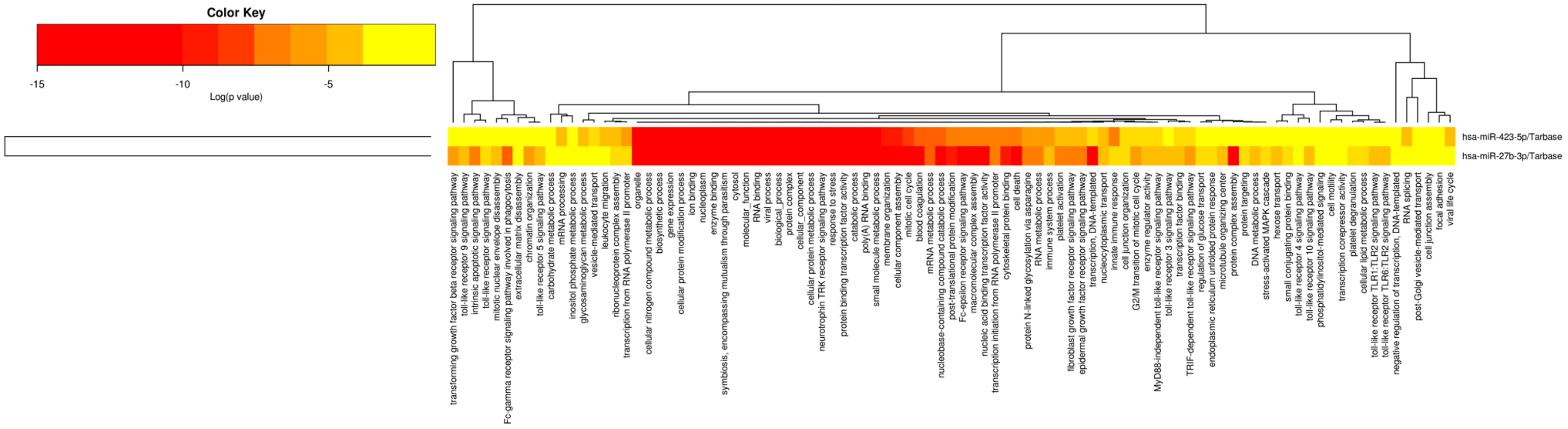
Color Key
-15
-10
-5
Log(p value)
hsa-miR-423-5p/Tarbase
hsa-miR-27b-3p/Tarbase
transforming growth factor beta receptor signaling pathway
toll-like receptor 9 signaling pathway
intrinsic apoptotic signaling pathway
toll-like receptor signaling pathway
mitotic nuclear envelope disassembly
Fc-gamma receptor signaling pathway involved in phagocytosis
extracellular matrix disassembly
chromatin organization
toll-like receptor 5 signaling pathway
carbohydrate metabolic process
mRNA processing
inositol phosphate metabolic process
glycosaminoglycan metabolic process
vesicle-mediated transport
leukocyte migration
ribonucleoprotein complex assembly
transcription from RNA polymerase II promoter
organelle
cellular nitrogen compound metabolic process
biosynthetic process
gene expression
cellular protein modification process
ion binding
nucleoplasm
enzyme binding
symbiosis, encompassing mutualism through parasitism
cytosol
molecular_function
RNA binding
viral process
biological_process
protein complex
cellular_component
cellular protein metabolic process
neurotrophin TRK receptor signaling pathway
response to stress
protein binding transcription factor activity
catabolic process
poly(A) RNA binding
small molecule metabolic process
membrane organization
cellular component assembly
mitotic cell cycle
blood coagulation
mRNA metabolic process
nucleobase-containing compound catabolic process
post-translational protein modification
Fc-epsilon receptor signaling pathway
macromolecular complex assembly
nucleic acid binding transcription factor activity
transcription initiation from RNA polymerase II promoter
cytoskeletal protein binding
cell death
protein N-linked glycosylation via asparagine
RNA metabolic process
immune system process
platelet activation
fibroblast growth factor receptor signaling pathway
epidermal growth factor receptor signaling pathway
transcription, DNA-templated
nucleocytoplasmic transport
innate immune response
cell junction organization
G2/M transition of mitotic cell cycle
enzyme regulator activity
MyD88-independent toll-like receptor signaling pathway
toll-like receptor 3 signaling pathway
transcription factor binding
TRIF-dependent toll-like receptor signaling pathway
regulation of glucose transport
endoplasmic reticulum unfolded protein response
microtubule organizing center
protein complex assembly
protein targeting
DNA metabolic process
stress-activated MAPK cascade
hexose transport
small conjugating protein binding
toll-like receptor 4 signaling pathway
toll-like receptor 10 signaling pathway
phosphatidylinositol-mediated signaling
cell motility
transcription corepressor activity
platelet degranulation
cellular lipid metabolic process
toll-like receptor TLR1:TLR2 signaling pathway
toll-like receptor TLR6:TLR2 signaling pathway
negative regulation of transcription, DNA-templated
RNA splicing
post-Golgi vesicle-mediated transport
cell junction assembly
focal adhesion
viral life cycle

Supplementary Table S1.

miRNA	p-value overall	FDR
**miR-124a**	**0.0003**	0.10
**miR-423-5p**	**0.0003**	0.61
**miR-127**	**0.0056**	0.62
**miR-27b-3p**	**0.0130**	0.81
**miR-483-5p**	**0.0136**	0.81
**miR-27b-5p**	**0.0196**	0.81
**miR-622**	**0.0197**	0.81
**miR-23a-5p**	**0.0217**	0.81
**miR-7-1-1**	**0.0221**	0.81

**miR-551b**	**0.0437**	0.87
**miR-532**	**0.0440**	0.87

FDR (false discovery rate) correction <0.2 and overall p-value <0.05 of the 11 miRNAs with significantly different expression levels among groups. Data were analyzed with a multiple linear regression model adjusted for BMI, pregestational-BMI, age, gestational age.
